# Different Neural Correlates of Emotion-Label Words and Emotion-Laden Words: An ERP Study

**DOI:** 10.3389/fnhum.2017.00455

**Published:** 2017-09-21

**Authors:** Juan Zhang, Chenggang Wu, Yaxuan Meng, Zhen Yuan

**Affiliations:** ^1^Faculty of Education, University of Macau Macau, China; ^2^Bioimaging Core, Faculty of Health Sciences, University of Macau Macau, China

**Keywords:** emotion-label word, emotion-laden word, N170, LPC, emotion effect

## Abstract

It is well-documented that both emotion-label words (e.g., sadness, happiness) and emotion-laden words (e.g., death, wedding) can induce emotion activation. However, the neural correlates of emotion-label words and emotion-laden words recognition have not been examined. The present study aimed to compare the underlying neural responses when processing the two kinds of words by employing event-related potential (ERP) measurements. Fifteen Chinese native speakers were asked to perform a lexical decision task in which they should judge whether a two-character compound stimulus was a real word or not. Results showed that (1) emotion-label words and emotion-laden words elicited similar P100 at the posteriors sites, (2) larger N170 was found for emotion-label words than for emotion-laden words at the occipital sites on the right hemisphere, and (3) negative emotion-label words elicited larger Late Positivity Complex (LPC) on the right hemisphere than on the left hemisphere while such effect was not found for emotion-laden words and positive emotion-label words. The results indicate that emotion-label words and emotion-laden words elicit different cortical responses at both early (N170) and late (LPC) stages. In addition, right hemisphere advantage for emotion-label words over emotion-laden words can be observed in certain time windows (i.e., N170 and LPC) while fails to be detected in some other time window (i.e., P100). The implications of the current findings for future emotion research were discussed.

## Introduction

Emotion is essential for the adaption and survival of human beings (Damasio, 2001). There are two widely explored dimensions including valence and arousal in existing emotion research (Lang et al., 1997). Valence is a bipolar dimension that categorizes emotions as positive (pleasure) or negative (displeasure), while arousal is a unipolar dimension that illustrates the degree to which emotion is activated in appetitive or defensive motivation system (Lang et al., 1997). To date, a growing body of research has employed emotion words as stimuli to investigate the complex cognitive mechanism underlying emotion language processing (Altarriba, 2006; Citron, 2012; Citron et al., 2014). Emotion words, regardless of valence, have been found to processed faster than neutral words and this phenomenon is known as emotion effect (Kousta et al., 2009; Vinson et al., 2014). Additionally, this effect is also accompanied with higher physiological activation for emotion words than neutral words (Citron, 2012).

However, recently, increasing attention has been paid to comparing “emotion words” and emotion-laden words (Pavlenko, 2008). “Emotion words” straightforwardly express particular affective states (e.g., happy, angry), whereas emotion-laden words (e.g., death, wedding) can arouse people's emotions without explicitly elucidating the affective states (Pavlenko, 2008; Altarriba and Basnight-Brown, 2011). Despite that it is worthwhile to differentiate the two types of words, it is ambiguous to use “emotion words” and emotion-laden words to distinguish emotion words, because broadly, emotion-laden words also belong to emotion words that are related to emotion, which is a common rationale in this field and would probably confuse the researchers who are not familiar with this argument. Therefore, a more precise approach to separate the two types of words is to refer to emotion-label words as directly demonstrating affective state and emotion-laden words as provoking people's emotion with connotation. We then relegate the emotion-label words and emotion-laden words to emotion words, because both of them are associated with emotion and can produce emotion activation (Kousta et al., 2009; Vinson et al., 2014).

One common manner of the previous emotion word research was that some researchers implicitly and unsystematically confused emotion-label words with emotion-laden words and considered them as one category (Ayçiçegi-Dinn and Caldwell-Harris, 2009; Kissler et al., 2009; Scott et al., 2009; Eilola and Havelka, 2011; Kousta et al., 2011; Min and Schirmer, 2011; Palazova et al., 2011; Yap and Seow, 2014; Chen et al., 2015). However, increasing behavioral studies have demonstrated that emotion-label words and emotion-laden words are different in producing emotion activation by using various experimental paradigms such as masked and unmasked priming tasks (Kazanas and Altarriba, 2015, 2016), affective Simon task (Altarriba and Basnight-Brown, 2011), and rapid serial visual presentation (RSVP) paradigm (Knickerbocker and Altarriba, 2013). For example, Knickerbocker and Altarriba (2013) compared the repetition blindness of emotion-label words, emotion-laden words, and neutral words by adopting the RVSP paradigm. In each trial of the task, a series of stimuli was sequentially and shortly (~75–125 ms) presented to the participant who was instructed to report the stimuli in order at the end of the trial. It was found that participants made more errors when reporting stimuli which had been repeated in the trial than when reporting those without repetition, which was known as repetition blindness (Kanwisher, 1987). In the study by Knickerbocker and Altarriba (2013), emotion-label words were found to produce larger repetition blindness compared to emotion-laden words and neutral words. Additional studies that used masked and unmasked priming tasks also showed that emotion-label words could be identified more quickly and generate a larger priming effect than emotion-laden words (Kazanas and Altarriba, 2015, 2016).

However, some other researchers argued that there was no difference between processing emotion-label words and emotion-laden words (Vinson et al., 2014; Martin and Altarriba, 2017). Martin and Altarriba (2017) compared the reaction times to emotion-label words and emotion-laden words in a lexical decision task and found that the two types of words were responded equally fast. Similarly, Kousta et al. (2009, 2011) claimed that emotion-label words and emotion-laden words (i.e., emotionally valenced words) were the same in emotion activation. By using the British Lexicon Project database (Keuleers et al., 2012), Vinson et al. (2014) demonstrated that both emotion-label words and emotion-laden words were processed faster than neutral words and the recognition speeds were similar for emotion-label words and emotion-laden words, even after controlling many cofounding variables such as word frequency and word familiarity. These findings suggested that the emotion activation was not only restricted to emotion words that explicitly describe emotion states (i.e., emotion-label words), but also was detected among emotion-laden words, and more importantly, the emotion activation was comparable between emotion-label words and emotion-laden words.

Taken together, it remains unclear whether emotion-label words and emotion-laden words can induce the same emotion activation given that the findings from previous behavioral studies are inconsistent. In contrast to behavior methods, neuroimaging techniques including event-related potentials (ERPs) have received extensive attentions in the investigation of the emotion processing of the brain (Citron, 2012). The ERP technology is sensitive to the time course of cognitive processing, offering the possibilities to resolve the enduring debate on the emotion activation of emotion-label words and emotion-laden words. Although, a large number of ERP studies have been performed to explore emotion words processing (Kissler et al., 2009; Scott et al., 2009; Sass et al., 2010; Palazova et al., 2011; Bayer et al., 2012; Yao and Wang, 2014; Zhang et al., 2014; Chen et al., 2015; Yi et al., 2015; Yao et al., 2016), none of them has investigated the possible differences underlying the cognitive mechanism of emotion-label words and emotion-laden words. In this study, ERP measurement was used to examine the neural responses of emotion-label words and emotion-laden words processing. The current study is of significant theoretical contribution to existing emotion research. By examining the different neural correlates of emotion-label words and emotion-laden words, it will help to deepen the understanding of the neural mechanism underlying emotion word recognition.

Three ERP components, including P100, N170, and Late Positive Complex (LPC), were relevant to our research goal according to previous studies (Hofmann et al., 2009; Scott et al., 2009; Sass et al., 2010; Palazova et al., 2011; Bayer et al., 2012; Zhang et al., 2014; Chen et al., 2015; Yi et al., 2015). Below, we briefly review each ERP component.

P100, a positivity usually peaking at around 100 ms after the stimulus onset, reflects attention bias toward emotion words (Hofmann et al., 2009; Sass et al., 2010) and is mostly detected at occipital sites. Zhang et al. (2014) discovered that the amplitude of occipital P100 was larger for negative words than for positive and neutral words by using the RSVP paradigm. By contrast, smaller P100 was identified for high frequency negative words than for high frequent positive and neutral words in an English lexical decision task (Scott et al., 2009). Although, the findings on the amplitude of P100 generated in the processing of positive and negative emotion words are inconsistent, one consensus has been reached that P100 is associated with emotion activation regardless of valence, such that emotion words elicit larger P100 than neutral words (Scott et al., 2009; Bayer et al., 2012).

N170 is a negative deflection peaking at around 170 ms after the stimulus onset over the occipital-temporal cortex. N170 is sensitive to differentiating emotional information and non-emotional information (Blau et al., 2007; Luo et al., 2010; Zhang et al., 2014). For example, Zhang et al. (2014) found that negative and positive emotion words generated larger N170 amplitude than neutral words at the occipital sites of the left hemisphere. In addition, many investigations of Chinese word recognition also concentrated on N170 and found that Chinese words reading could elicit N170 over occipito-temporal sites on both left hemisphere (Lee et al., 2007; Lin et al., 2011) and right hemisphere (Lee et al., 2007; Wang et al., 2011).

The last target ERP component, LPC (Late Positive Complex), usually peaks between 500 and 700 ms at the central sites and is modulated by the valence of the stimulus, reflecting a sustained elaborate processing of emotional stimuli (Citron, 2012; Zhang et al., 2014). Although, some previous work revealed that positive words elicited larger LPC amplitude as compared to neutral words and negative words (Herbert et al., 2008; Kissler et al., 2009; Zhang et al., 2014), some other contradictory evidence revealed that negative words evoked larger LPC than positive words and neutral words (Bernat et al., 2001; Kanske and Kotz, 2007). These inconsistent results may be resulted from many factors such as task demands, materials, and individual differences (Citron, 2012). Regardless of this controversy, LPC is generally considered to be sensitive to the valence of emotion words (Citron, 2012).

Founded on robust findings of ERP correlates of emotion words (i.e., P100, N170, and LPC), the current study aims to investigate the neural responses to the processing of emotion-label words and emotion-laden words. More specifically, since emotion-label words explicitly label emotion states while emotion-laden words only implicitly express emotional states by connotation (Pavlenko, 2008), it is hypothesized that emotion-label words may elicit larger P100, N170, and LPC than emotion-laden words. The valence effect is not direct to predict, because findings about valence effect are inconsistent (Citron, 2012). As P100 is associated with early attention allocation and negative bias allows individual to cope with dangerous conditions (Zhang et al., 2014), it is predicted that negative words will evoke larger P100 than positive words. Since N170 reflects a discrimination of emotion stimuli and neutral stimuli, positive words and negative words will probably elicit similar N170. Finally, LPC is widely believed to associate with valence, regardless of the mixed findings on this notion. Zhang et al. (2014) argued that LPC distinguished the positive and negative words because mounting evidence showed that positive words generated larger LPC than negative words, accompanied with a negative bias in early stages (e.g., larger P100 was elicited by negative words than by positive words). Therefore, it is hypothesized that positive words will elicit larger LPC than negative words, reflecting a “positive offset” that pleasant word enhances elaborating process in late stage (Zhang et al., 2014).

## Methods

### Participants

Sixteen right-handed healthy college students recruited from the University of Macau (aged 21–31 years, *M* = 24.09, *SD* = 2.95, 9 males) participated in this study. All of the participants were required to sign informed consent documents prior to the experiment. The protocol for the experimental trial was approved by the Institutional Review Board in the University of Macau. The participants received 7.5 dollars per hour as compensation for their participation. All participants were Chinese native speakers with normal or corrected-to-normal vision. Participants had no history of neurobiological or psychiatric disorders and all reported they were right-handed. Data from one participant were discarded due to the exceedingly high artifacts of EEG data. Consequently, the recordings from the remaining fifteen participants (8 males; mean age 23.87 years, *SD* = 3.11; age range 20–31 years) were included for further analyses.

### Stimuli

Experimental materials include a total of 60 two-character Chinese emotion-label words and 60 two-character Chinese emotion-laden words. Within the emotion-label words, 30 were negative words and the other 30 were positive ones. Similarly, 60 emotion-laden words consisted of 30 positive words and 30 negative ones. All of the words were chosen from SUBTLEX-CH (Cai and Brysbaert, 2010), a comprehensive Chinese word database. To ensure the valence and balance the arousal levels between emotion-label words and emotion-laden words, the valence and arousal levels of the 120 words were rated by another group of 20 participants on a 7-point Likert scale. Finally, the four types of words were matched on strokes [*F*_(3, 116)_ = 2.125, *p* > 0.1], word frequency [*F*_(3, 116)_ = 0.069, *p* > 0.9], and arousal levels [*F*_(3, 116)_ = 0.142, *p* > 0.9]. In addition, negative words were rated to be more negatively than positive words [*F*_(3, 116)_ = 340.604, *p* < 0.001], while emotion-label word and emotion-laden words were matched on valence (*ps* > 0.1, see Table 1 on the stimulus properties). In order to balance the yes and no responses in the lexical decision task, another 120 Chinese pseudowords were produced by randomly combining two Chinese real characters. Chinese pseudowords (mean strokes: 16.00) and Chinese words (mean strokes: 16.91) were not different on strokes [*F*_(1, 238)_ = 3.09, *p* = 0.08].

**Table 1:** Means (M) and Standard Deviation (SD) in brackets for stimulus properties for emotion words and emotion-laden words.

	**Emotion-label words**		**Emotion-laden words**	
	**Negative**	**Positive**	**Negative**	**Positive**
Valence	2.67 (0.37)	5.24 (0.40)	2.39 (0.41)	5.08 (0.54)
Frequency (logW)	2.34 (0.84)	2.35 (0.89)	2.39 (0.66)	2.34 (0.69)
Strokes	16.63 (3.40)	18.13 (3.17)	16.03 (3.80)	16.83 (4.61)
Arousal	4.81 (0.27)	4.83 (0.29)	4.87 (0.32)	4.85 (0.57)

### Procedures

**Figure 1:**
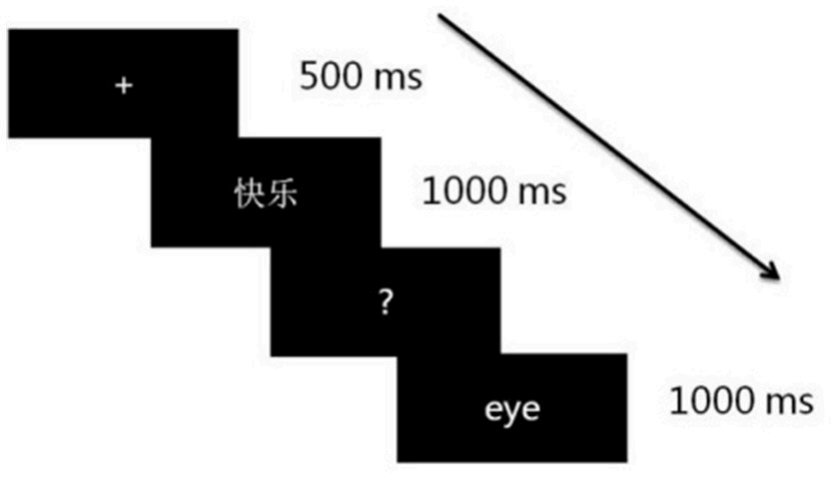
Scheme of one experimental trial.

Stimuli were presented in white characters (font: Song typeface, size: 48 points) on a black background in the center of a 19-in. monitor. During the experiment, the participant was seated on a comfortable chair in a sound-attenuated room. The distance between the participant and the computer screen was around 70 cm. The whole experiment consisted of seven blocks, including one training block and six experimental blocks. The training block aimed to familiarize participants with the experimental procedure and it included 12 pseudowords and 12 two-character Chinese words with three words being in each category (i.e., negative emotion-label words, positive emotion-label words, negative emotion-laden words, and positive emotion-laden words). None of the training materials was included in the formal experimental blocks. Each experimental block contained 80 trials including 40 Chinese words (ten words for each category) and 40 Chinese pseudowords. Each Chinese word was presented to participants twice in total through two different blocks. As depicted in Figure 1, each trial started with a 500 ms fixation cross on the center of the screen, followed by the target item that lasted for 1,000 ms. During the presentation of the target item, participants were instructed not to respond until the item was replaced by a question mark. When participants saw the question mark, they should judge whether the target item was a Chinese word or not as quickly and accurately as possible. Each trial ended with an eye that suggested participants could blink their eyes if they wanted. Altogether, there were 480 trials distributed in the 6 formal experimental blocks. The order of blocks and the order of trials within one block were randomly sequenced. The experimental procedure was programmed with E-prime 2.0.

### EEG recording and processing

The electroencephalogram (EEG) data were recorded from 128 scalp sites by using a Hydro Cel Geodesic Sensor Net (Electrical Geodesics, Inc. EGI; Eugene, OR) with a sampling rate of 1,000 Hz. The recording was conducted in a sound-proofed experimental room. The impedances of all electrodes were kept below 50 kΩ during the whole experiment. The data were processed off-line with EGI Net Station Waveform Tools and were first filtered with a 0.1–30 Hz band pass. Then, data segmentation surrounding the trigger onset time points was performed (i.e., from −100 ms prior to the trigger onset until 800 ms after the trigger onset). Automatic rejection criteria were applied to mark contaminated segments. Specifically, voltage variation of more than 70 μV was regarded as eye blink and trials with signals exceeding ±27.5 μV were treated as eye movement. Artifact detection was also performed manually to exclude contaminated epochs. In this study, trials that had more than 10% channels labeled as artifacts or contemned by blinks or eye movements were discarded for further analysis. In this way, around 25% trials were deleted with at least 40 trials per condition being kept for analysis. For each participant and each condition, ERP waveforms were corrected relative to a 100 ms prestimulus baseline and were averaged.

**Figure 2:**
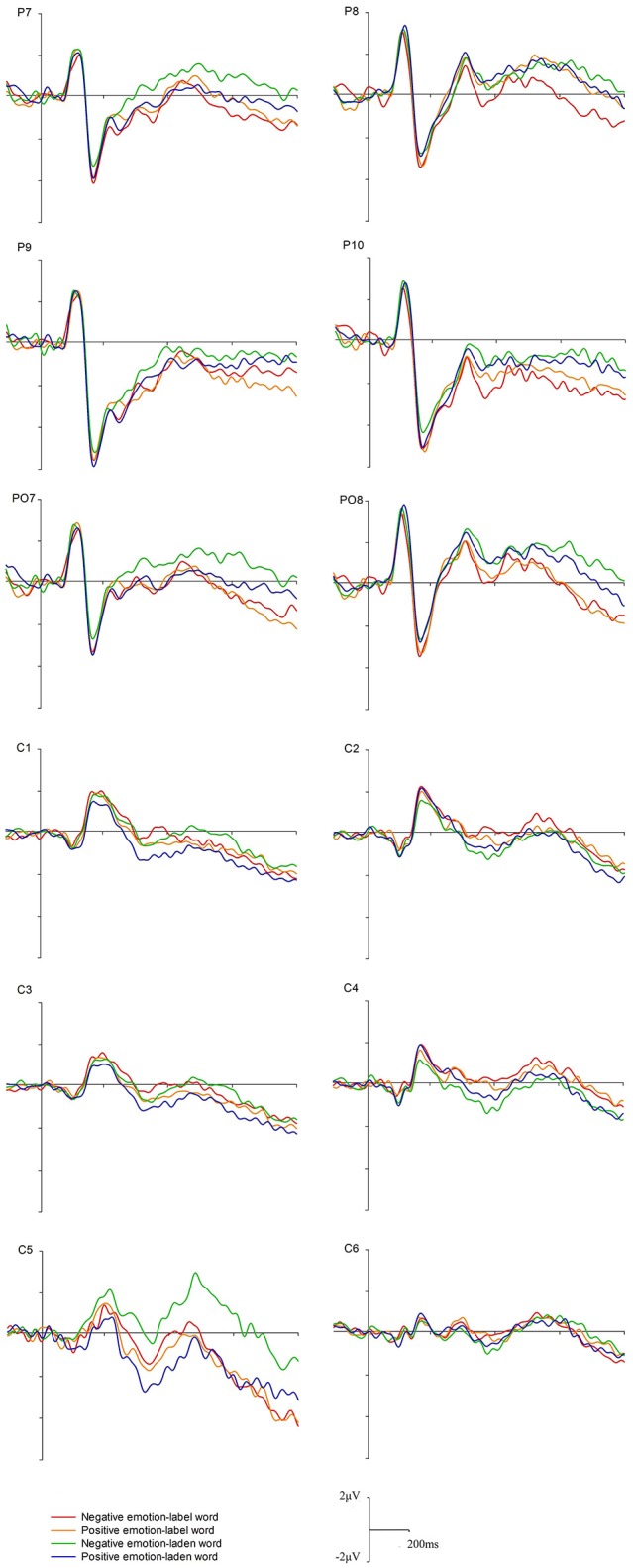
Grand average ERPs of the P100, N170, and LPC components at the indicated electrode sites.

**Figure 3:**
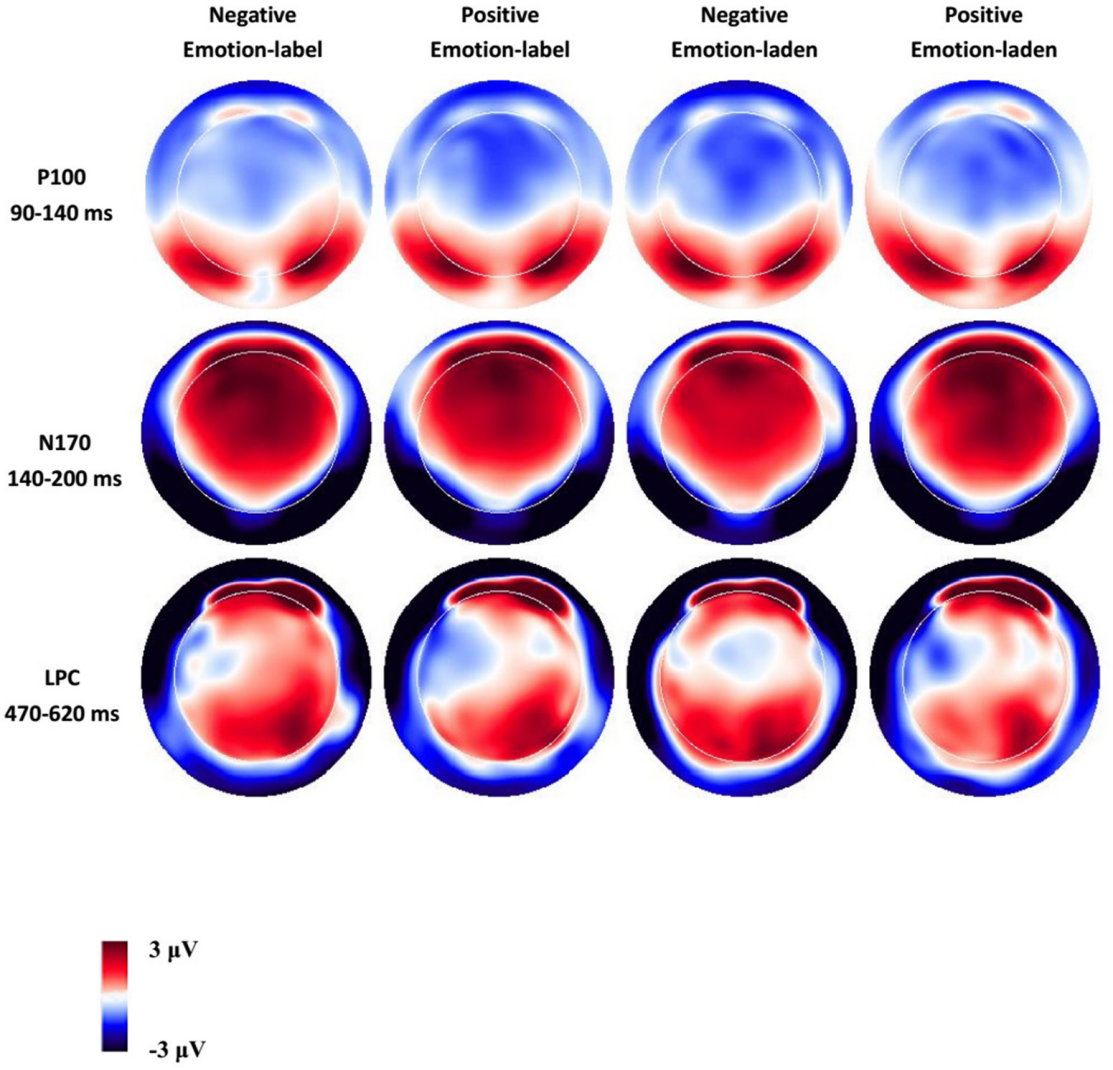
Grand average ERP topographies of the P100, N170, and LPC components across four word types.

Based on the visual inspection of the brain topography (Figure 2), grand-averaged waveforms (Figure 3), and prior investigations (Zhang et al., 2014; Chen et al., 2015), we analyzed three obvious ERP components including P100, N170, and LPC within the time windows of 90–140, 140–200, and 470–620, respectively. For P100 and N170, the mean amplitude of electrodes in the occipital area (P7/P8, P9/P10, and PO7/PO8) along both hemispheres were calculated, in alignment with previous studies (Scott et al., 2009; Bayer et al., 2012). In addition, the mean amplitude of the central cites (CP1/CP2, CP3/CP4, and CP5/CP6) within the 470–620 ms time window (LPC) was analyzed for LPC. Statistical significance was performed through a Greenhouse-Geisser adjustment at the level of 0.05.

## Results

The analysis of behavior data was not performed due to the high accuracy rate (96.11% for all participants) and the fixed duration of target word presentation. Therefore, we mainly focused on ERP data analysis to identify the significant neural markers underlying the processing of emotion-label words and emotion-laden words recognition. Figures 2, 3 display the ERP waveforms for each condition at indicated electrodes (i.e., P9/10, C3/4). Clearly, P100 and N170 components at the occipital-temporal sites and LPC at the central sites were identified.

### P100 (90–140 ms)

A 2 (word type: emotion-label words, emotion-laden words) × 2 (valence: negative, positive) × 3 (electrode: P7/P8, P9/P10, PO7/PO8) × 2 (hemisphere: left, right) repeated-measures ANOVA was conducted. No main effects for word type, valence, electrodes, hemisphere, or other interactions among those factors were observed. A significant interaction between electrodes and hemisphere [*F*_(2, 28)_ = 4.82, *p* < 0.05, η^2^ = 0.26] was discovered. Further comparisons showed no significant differences among the six electrodes over two hemispheres (*ps* > 0.05).

### N170 (140–200 ms)

Similar with P100, a 2 (word type: emotion-label words, emotion-laden words) × 2 (valence: negative, positive) × 3 (electrode: P7/P8, P9/P10, PO7/PO8) × 2 (hemisphere: left, right) repeated-measures ANOVA was conducted for N170. The results showed a significant main effect of electrodes [*F*_(2, 28)_ = 16.92, *p* < 0.001, η^2^ = 0.55]. *Post-hoc* comparison exhibited that larger N170 were elicited in P9/P10 than in P7/P8 and PO7/PO8 [*t*_(14)_ = 3.70, *p* = 0.002, *t*_(14)_ = 5.43, *p* < 0.001]. More importantly, there was a significant interaction between word type and hemisphere [*F*_(1, 14)_ = 5.48, *p* < 0.05, η^2^ = 0.28]. *Post-hoc* comparison revealed that on the right hemisphere, emotion-label words elicited larger N170 than emotion-laden words [*t*_(14)_ = 2.38, *p* < 0.05]. However, this was not the case for the left hemisphere where emotion-label words and emotion-laden words evoked similar N170 (*ts* < 1).

### LPC (470–620 ms)

Finally, a 2 (word type: emotion-label words, emotion-laden words) × 2 (valence: negative, positive) × 3 (electrode: C1/C2, C3/C4, C5/C6) × 2 (hemisphere: left, right) repeated-measures ANOVA was conducted for LPC. There was a main effect of valence, with negative words producing larger LPC than positive words [*F*_(1, 14)_ = 4.81, *p* < 0.05, η^2^ = 0.26]. In addition, a three-way interaction of word type, valence, and hemisphere was also identified [*F*_(1, 14)_ = 6.65 *p* = 0.02, η^2^ = 0.32]. *Post-hoc* comparison showed that negative emotion-label words elicited larger LPC on the right hemisphere than on the left hemisphere [*t*_(14)_ = 2.48, *p* < 0.05], while other comparisons failed to reveal significant differences (*ps* > 0.05).

## Discussion

The primary aims of the present study were to examine the cortical responses to emotion-label words and emotion-laden words and to explore whether the neural correlates of the two kinds of words exhibit any significant differences. To achieve these goals, fifteen Chinese speakers performed a lexical decision task with ERPs measured. The target stimuli included positive emotion-label words, negative emotion-label words, positive emotion-laden words, and negative emotion-laden words. Overall, we identified three emotion processing related ERP components: P100 (Herbert et al., 2008; Sass et al., 2010; Bayer et al., 2012), N170 (Scott et al., 2009; Frühholz et al., 2011; Zhang et al., 2014), and LPC (Bernat et al., 2001; Frühholz et al., 2011; Zhang et al., 2014). Differences between emotion-label words and emotion-laden words were observed in N170, and LPC, although it was not the case for P100.

The present study showed that both emotion-label words and emotion-laden words, regardless of valence, could elicit similar P100 at the posterior sites. The result indicated that emotion-label words and emotion-laden words captured similar attention allocation in pre-attention stage. The result that there was no valence effect was inconsistent with previous studies (Bayer et al., 2012; Zhang et al., 2014). For example, Bayer et al. (2012) found P100 in both the silent reading and lexical decision tasks and P100 elicited by positive emotion words was found to be larger than that by negative emotion words. By contrast, Zhang et al. (2014) demonstrated that P100 was stronger for negative words than for neutral words and positive words, indicating that P100 might reflect a discrimination of threatening and non-threatening information. However, the identified P100 in the present work failed to exhibit significant valence difference (Scott et al., 2009; Bayer et al., 2012; Zhang et al., 2014). The inconsistent findings in this processing stage require future research to investigate valence effect on P100.

As for N170, in the present study, we discovered that larger N170 was elicited by emotion-label words than by emotion-laden words, although the difference was only identified on the right hemisphere. At first, N170 was considered to be associated with emotional face recognition (Luo et al., 2010; Frühholz et al., 2011), which usually peaked at around 170 ms after stimuli onset with a posterior scalp distribution. Recently, it has been revealed that that N170 could also be elicited by emotion words. For example, Zhang et al. (2014) found that negative words and positive words elicited larger N170 than neutral words, intensively at posterior sties on the left hemisphere and argued that the N170 was associated with emotion effect and reflected the discrimination of emotional and non-emotional information. This result echoed one of the current findings that emotion-label words and emotion-laden words elicited similar N170 on the left hemisphere. However, there could be a difference in the activation levels between the two types of words on the right hemisphere because of the following two reasons. First, emotion-label words could explicitly and directly elicit emotion states while emotion-laden words express emotion implicitly. Therefore, it might be easier to provoke emotion for emotion-label words than for emotion-laden words. Second, previous studies have suggested that the right hemisphere has the advantage of emotion perception and expression over the left hemisphere (Borod et al., 1998; Smith and Bulman-Fleming, 2005). Thus, the difference in emotion activation may be more detectable on the right than on the left hemisphere. Finally, a large body of investigations demonstrated that the amplitude of N170 in word recognition was affected by attention (Lee et al., 2007; Yoncheva et al., 2010, 2015; Zhao et al., 2012). Therefore, it was possible that the differences between emotion-label words and emotion-laden words were attributed to enhanced attention that was captured by the emotion-label words.

As for the final ERP component LPC, a late positivity (usually 500–600 ms after stimuli onset) at the central sites (Citron, 2012) reflecting a late and deeper elaboration of focused information (Citron, 2012; Chen et al., 2015), two main findings were revealed in the present study. First, a main effect of valence was identified that negative words generated larger LPC than positive words. The result was in line with previous findings that negative words induced larger LPC than neutral words and positive words (Bernat et al., 2001; Kanske and Kotz, 2007). However, there was some evidence that indicated negative words elicited larger LPC than positive words (Herbert et al., 2008; Kissler et al., 2009; Zhang et al., 2014). Zhang et al. (2014) attributed such positivity bias to “positivity offset” that was responsive to processing priority for negative words at early stages and positive words therefore enhance elaboration at later stages. By contrast, in the present study, we found negative bias on LPC. There might be two reasons to explain our results. First reason was that we not only included emotion-laden words but also emotion-label words. Possibly, a late positivity bias could be associated with large proportion of emotion-laden words in the stimuli list. After increasing the number of emotion-label words, a negative bias would probably be expected. For example, Bernat et al. (2001) found negative emotion-label words elicited larger LPC than positive emotion-label words and Bernat et al. (2001) did not contain any emotion-laden words and only included emotion-label words. Secondly, we did not find negative bias at pre-attention stage as Zhang et al. (2014) did. Therefore, the finding of no positivity bias in LPC was probably due to no negative bias being observed at first and no “positivity offset” was identified consequently. Of course, these exploratory predictions require further research testing.

Second important finding in LPC was a three-way interaction and *post-hoc* comparison showed that larger LPC was identified for negative emotion-label words on the right hemisphere than that on the left hemisphere, whereas no hemisphere difference was found for positive emotion-label words and emotion-laden words. This finding could be explained from the perspective that the right hemisphere advantage was exclusively restricted to negative emotions (Najt et al., 2013). More specifically, Najt et al. (2013) used visual half field (VHF) technique to investigate the lateralization of facial emotion perception. Participants were instructed to indicate as quickly and accurately as possible whether faces that were presented in left visual field (LVF/right hemisphere) or in right visual field (RVF/left hemisphere) were neutral or emotional. The results revealed that negative emotional faces (e.g., sadness, fear, and anger) were recognized more accurately when they were presented in the LVF than when presented in the RVF, suggesting a right hemisphere advantage for negative emotional faces. However, there was no difference between two hemispheres for positive emotional faces. Our results further confirmed that the right hemisphere advantage might be exclusively restricted to negative emotion stimuli that included emotional faces (Najt et al., 2013) and emotion-label words but not emotion-laden words.

Although, the present study demonstrated that emotion-label words and emotion-laden words were different in N170 and LPC, indicating distinct neural responses of the two types of words. Some contradictory behavioral data suggested that emotion-label words and emotion-laden words were identified similarly (Martin and Altarriba, 2017). One reason for the non-significant difference between the two types of words might be that it was random presentation of emotion-label words and emotion-laden words in one block that reduced the possible differences between them, as argued by Martin and Altarriba (2017). However, in the present study, following Martin and Altarriba (2017) who mixed emotion-label words and emotion-laden words in one block and presented the words randomly, we still found a significant difference between emotion-label words and emotion-laden in N170 and LPC. Therefore, high sensitivity and temporal resolution of ERP allowed us to examine the precise distinctions between emotion-label words and emotion-laden words.

Some contradictory argument (Kousta et al., 2011; Vinson et al., 2014) has claimed the emotion activation was similar in emotion-label words and emotion-laden words. By contrast, the present study showed that there was a difference in terms of the extent to which emotion activation was induced by emotion-label words and emotion-laden. On the right hemisphere where emotion processing was more targeted, N170 was elicited larger by emotion-label words than emotion-laden words. Additionally, negative emotion-label words evoked larger LPC on the right hemisphere than on the left hemisphere, while there was no difference between two hemispheres for emotion-laden words. These results indicated that emotion-label words might generate larger emotion activation than emotion-laden words. Therefore, it is necessary to separate emotion-label words and emotion-laden words apart when constructing a stimuli list in future emotion word research. However, note also that no neutral words were included in the present study because our main purpose was to compare emotion-label words and emotion-laden words. This could be a limitation that we were not able to compare emotion-label words and emotion-laden words against neutral words. Future studies could include neutral words to compare emotion effect between emotion-label words and emotion-laden words.

## Conclusion

In summary, the present study found that emotion-label words elicited larger N170 than emotion-laden words on the right hemisphere. In addition, emotion-label words (especially negative emotion-label words) elicited larger LPC on the right hemisphere than on the left hemisphere. These results support that emotion-label words and emotion-laden words are processed distinctively at different stages. Hence, it is highly suggested that future emotion research should consider separating emotion-label words and emotion-laden words when using words as emotional stimuli, given that previous studies (Altarriba and Basnight-Brown, 2011; Knickerbocker and Altarriba, 2013; Kazanas and Altarriba, 2015, 2016), along with the current study, have revealed that emotion-label words and emotion-laden words are different in cognitive processing and neural correlates.

## Author contributions

JZ and CW developed the research idea and designed the study. CW conducted the experiment and analyzed the data. JZ and CW wrote the manuscript. YM and ZY reviewed and revised the manuscript.

### Conflict of interest statement

The authors declare that the research was conducted in the absence of any commercial or financial relationships that could be construed as a potential conflict of interest.

## Supplementary material

Supplementary Figure 1 Grand average ERPs of the P100, N170, and LPC components at the indicated electrode sites with pseudowords added.
