# Corrigendum: Different Neural Correlates of Emotion-Label Words and Emotion-Laden Words: An ERP Study

**DOI:** 10.3389/fnhum.2017.00589

**Published:** 2017-11-29

**Authors:** Juan Zhang, Chenggang Wu, Yaxuan Meng, Zhen Yuan

**Affiliations:** ^1^Faculty of Education, University of Macau, Macau, China; ^2^Bioimaging Core, Faculty of Health Sciences, University of Macau, Macau, China

**Keywords:** emotion-label word, emotion-laden word, N170, LPC, emotion effect

In the original article, there was an error. At page 4, we wrote wrong electrodes, the centrals sites should be C1/C2, C3/C4, C5/C6 instead of CP1/CP2, CP3/CP4, and CP5/CP6, making it coherent with other parts of the paper.

A correction has been made to Method, EEG Recording and Processing, Paragraph 2: Based on the visual inspection grand-averaged waveforms (Figure 2), brain topography (Figure 3), and prior investigations (Zhang et al., 2014; Chen et al., 2015), we analyzed three obvious ERP components including P100, N170, and LPC within the time windows of 90–140, 140–200, and 470–620, respectively. For P100 and N170, the mean amplitude of electrodes in the occipital area (P7/P8, P9/P10, and PO7/PO8) along both hemispheres were calculated, in alignment with previous studies (Scott et al., 2009; Bayer et al., 2012). In addition, the mean amplitude of the central cites (C1/C2, C3/P4, and C5/C6) within the 470–620 ms time window (LPC) was analyzed for LPC. Statistical significance was performed through a Greenhouse-Geisser adjustment at the level of 0.05.

In the original article, there was an error. At page 4, we indicated the wrong Figure with text, the brain topography should be Figure 3, while grand-averaged waveforms should be Figure 2. In the text, we used the opposite reference.

A correction has been made to Method, EEG Recording and Processing, Paragraph 2: Based on the visual inspection of the grand-averaged waveforms (**Figure 2**), brain topography (**Figure 3**), and prior investigations (Zhang et al., 2014; Chen et al., 2015), we analyzed three obvious ERP components including P100, N170, and LPC within the time windows of 90–140, 140–200, and 470–620, respectively. For P100 and N170, the mean amplitude of electrodes in the occipital area (P7/P8, P9/P10, and PO7/PO8) along both hemispheres were calculated, in alignment with previous studies (Scott et al., 2009; Bayer et al., 2012). In addition, the mean amplitude of the central cites (C1/C2, C3/P4, and C5/C6) within the 470–620 ms time window (LPC) was analyzed for LPC. Statistical significance was performed through a Greenhouse-Geisser adjustment at the level of 0.05.

In the original article, there was an error. At page 4, in Result part, we misused “i.e.,”, and a better way is to say all of the electrodes in the Figure 2.

A correction has been made to Result, Paragraph 1: The analysis of behavior data was not performed due to the high accuracy rate (96.11% for all participants) and the fixed duration of target word presentation. Therefore, we mainly focused on ERP data analysis to identify the significant neural markers underlying the processing of emotion-label words and emotionladen words recognition. Figures 2,3 display the ERP waveforms for each condition at indicated electrodes (i.e., P7/P8, P9/P10, PO7/PO8, C1/C2, C3/C4, and C5/C6). Clearly, P100 and N170 components at the occipital-temporal sites and LPC at the central sites were identified.

In the original article, there was an error. At page 6, in Discussion part, we made a wrong statement that misconnected the sentences. Original sentence stated that however, negative emotion words elicited larger LPC than positive words. But previous sentence conveyed the same message as the sentence did. Apparently, here, the “however” was misused and the sentence should be corrected, because it would confuse the readers.

A correction has been made to Discussion, Paragraph 4: As for the final ERP component LPC, a late positivity (usually 500–600 ms after stimuli onset) at the central sites (Citron, 2012) reflecting a late and deeper elaboration of focused information (Citron, 2012; Chen et al., 2015), two main findings were revealed in the present study. First, a main effect of valence was identified that negative words generated larger LPC than positive words. The result was in line with previous findings that negative words induced larger LPC than neutral words and positive words (Bernat et al., 2001; Kanske and Kotz, 2007). However, there was some evidence that indicated positive words elicited larger LPC than negative words (Herbert et al., 2008; Kissler et al., 2009; Zhang et al., 2014). Zhang et al. (2014) attributed such positivity bias to “positivity offset” that was responsive to processing priority for negative words at early stages and positive words therefore enhance elaboration at later stages. By contrast, in the present study, we found negative bias on LPC. There might be two reasons to explain our results. First reason was that we not only included emotion-laden words but also emotion-label words. Possibly, a late positivity bias could be associated with large proportion of emotion-laden words in the stimuli list. After increasing the number of emotion-label words, a negative bias would probably be expected. For example, Bernat et al. (2001) found negative emotion-label words elicited larger LPC than positive emotion-label words and Bernat et al. (2001) did not contain any emotion-laden words and only included emotion-label words. Secondly, we did not find negative bias at pre-attention stage as Zhang et al. (2014) did. Therefore, the finding of no positivity bias in LPC was probably due to no negative bias being observed at first and no “positivity offset” was identified consequently. Of course, these exploratory predictions require further research testing.

In the original article, there was an error. At page 7, we were short of a word. Specifically, we used emotion-laden instead of emotion-laden words. The “word” was apparently missing.

A correction has been made to Discussion, Paragraph 7: Some contradictory argument (Kousta et al., 2011; Vinson et al., 2014) has claimed the emotion activation was similar in emotion-label words and emotion-laden words. By contrast, the present study showed that there was a difference in terms of the extent to which emotion activation was induced by emotion-label words and emotion-laden words. On the right hemisphere where emotion processing was more targeted, N170 was elicited larger by emotion-label words than emotion-laden words. Additionally, negative emotion-label words evoked larger LPC on the right hemisphere than on the left hemisphere, while there was no difference between two hemispheres for emotion-laden words. These results indicated that emotion-label words might generate larger emotion activation than emotion-laden words. Therefore, it is necessary to separate emotion-label words and emotion-laden words apart when constructing a stimuli list in future emotion word research. However, note also that no neutral words were included in the present study because our main purpose was to compare emotion-label words and emotion-laden words. This could be a limitation that we were not able to compare emotion-label words and emotion-laden words against neutral words. Future studies could include neutral words to compare emotion effect between emotion-label words and emotion-laden words.

The authors apologize for these errors and state that this does not change the scientific conclusions of the article in any way.

## Conflict of interest statement

The authors declare that the research was conducted in the absence of any commercial or financial relationships that could be construed as a potential conflict of interest.

## References

[B1] Bayer M. Sommer W. Schacht A. (2012). P1 and beyond: functional separation of multiple emotion effects in word recognition. Psychophysiology 49, 959–969. 10.1111/j.1469-8986.2012.01381.x 22594767

[B2] Bernat E. Bunce S. Shevrin H. (2001). Event-related brain potentials differentiate positive and negative mood adjectives during both supraliminal and subliminal visual processing. Int. J. Psychophysiol. 42, 11–34. 10.1016/S0167-8760(01)00133-7 11451477

[B3] Chen P. Lin J. Chen B. Lu C. Guo T. (2015). Processing emotional words in two languages with one brain: ERP and fMRI evidence from Chinese–English bilinguals. Cortex 71, 34–48. 10.1016/j.cortex.2015.06.002 26143622

[B4] Citron F. M. (2012). Neural correlates of written emotion word processing: a review of recent electrophysiological and hemodynamic neuroimaging studies. Brain Lang. 122, 211–226. 10.1016/j.bandl.2011.12.007 22277309

[B5] Herbert C. Junghofer M. Kissler J. (2008). Event related potentials to emotional adjectives during reading. Psychophysiology 45, 487–498. 10.1111/j.1469-8986.2007.00638.x 18221445

[B6] Kanske P. Kotz S. A. (2007). Concreteness in emotional words: ERP evidence from a hemifield study. Brain Res. 1148, 138–148. 10.1016/j.brainres.2007.02.044 17391654

[B7] Kissler J. Herbert C. Winkler I. Junghofer M. (2009). Emotion and attention in visual word processing—an ERP study. Biol. Psychol. 80, 75–83. 10.1016/j.biopsycho.2008.03.004 18439739

[B8] Kousta S.-T. Vigliocco G. Vinson D. P. Andrews M. Del Campo E. (2011). The representation of abstract words: why emotion matters. J. Exp. Psychol. Gen. 140, 14–34. 10.1037/a0021446 21171803

[B9] Scott G. G. O'Donnell P. J. Leuthold H. Sereno S. C. (2009). Early emotion word processing: evidence from event-related potentials. Biol. Psychol. 80, 95–104. 10.1016/j.biopsycho.2008.03.010 18440691

[B10] Vinson D. Ponari M. Vigliocco G. (2014). How does emotional content affect lexical processing? Cogn. Emot. 28, 737–746. 10.1080/02699931.2013.851068 24215294 PMC3979450

[B11] Zhang D. He W. Wang T. Luo W. Zhu X. Gu R.. (2014). Three stages of emotional word processing: an ERP study with rapid serial visual presentation. Soc. Cogn. Affect. Neurosci. 9, 1897–1903. 10.1093/scan/nst188 24526185 PMC4249467

