# Supplementary figures and images for: Different Neural Correlates of Emotion-Label Words and Emotion-Laden Words: An ERP Study
Source: Front Hum Neurosci. 2017 Sep 21;11:455. doi: 10.3389/fnhum.2017.00455 (PMC5613167; doi:10.3389/fnhum.2017.00455)

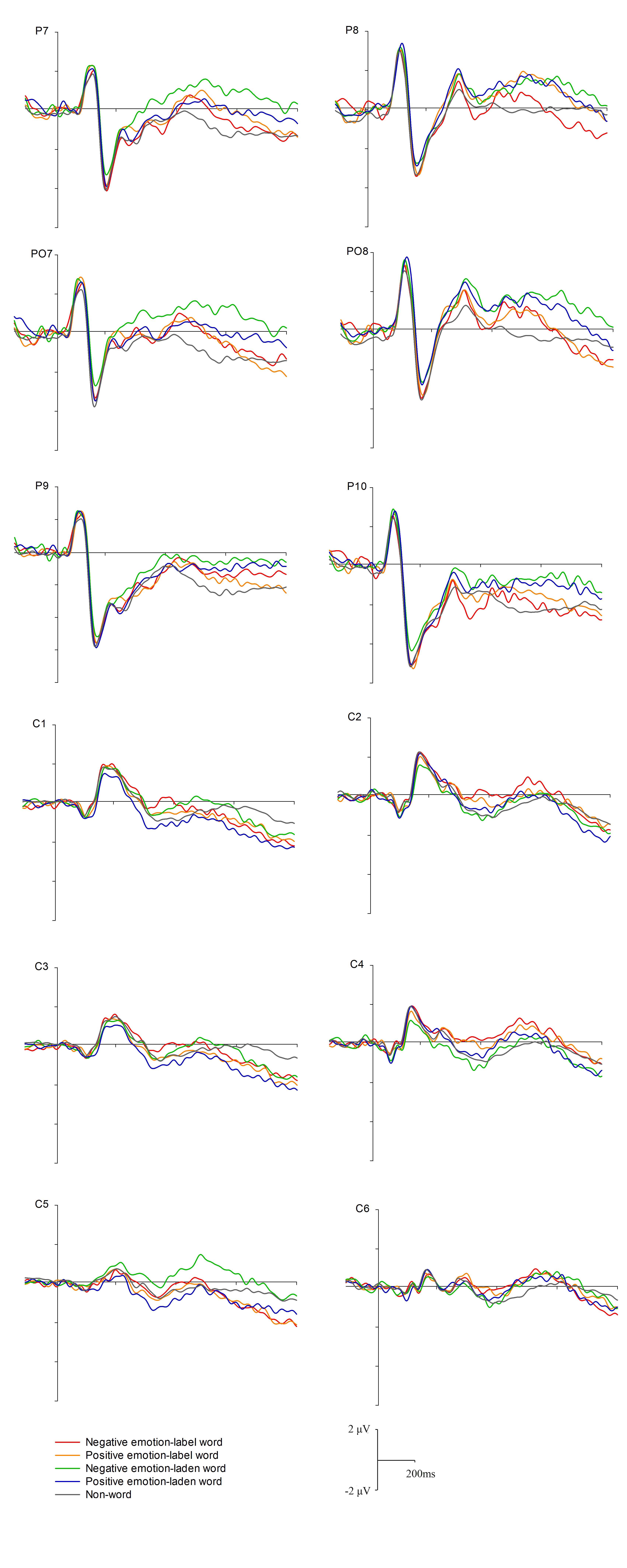

Supplement: Supplementary Figure 1 — Grand average ERPs of the P100, N170, and LPC components at the indicated electrode sites with pseudowords added. [file Image1.JPEG]
